# Primary Prevention of Intimate Partner Violence Among Recently Married Dyads Residing in the Slums of Pune, India: Development and Rationale for a Dyadic Intervention

**DOI:** 10.2196/11533

**Published:** 2019-01-18

**Authors:** Ameeta Shivdas Kalokhe, Sandhya Iyer, Tuman Katendra, Keshav Gadhe, Ambika R Kolhe, Anuradha Paranjape, Carlos del Rio, Rob Stephenson, Seema Sahay

**Affiliations:** 1 Division of Infectious Diseases Department of Medicine Emory University School of Medicine Atlanta, GA United States; 2 Department of Global Health Emory Rollins School of Public Health Atlanta, GA United States; 3 Department of Social and Behavioral Research National AIDS Research Institute Indian Council of Medical Research Pune India; 4 Department of Medicine Temple University Lewis Katz School of Medicine Philadelphia, PA United States; 5 Department of Systems, Populations and Leadership University of Michigan School of Nursing Ann Arbor, MI United States

**Keywords:** intimate partner violence, prevention, gender-based violence, domestic violence, intervention

## Abstract

**Background:**

Intimate partner violence (IPV) is frequently experienced by women of low socioeconomic status in India. It is a human rights violation and associated with negative effects on physical and mental well-being, underscoring the need for effective prevention strategies.

**Objective:**

This study aimed to develop a dyadic intervention for the primary prevention of IPV among newly married couples residing in slum communities in India.

**Methods:**

The intervention was developed using a community-based, mixed-methods design rooted in couple-interdependence theory and guided by the intervention mapping (IM) framework. It used the six critical IM steps to inform the content and delivery of the intervention: (1) needs assessment, (2) preparation of matrices of change objectives, (3) selection of theory-based methods and practical applications, (4) production of intervention components and materials, (5) intervention adoption and implementation, and (6) evaluation planning.

**Results:**

The resulting *Ghya Bharari Ekatra* (*Take a Flight Together*) intervention is intended to be delivered in 6 weekly sessions by a trained pair of male and female lay community educators to groups of 3 to 5 newly married couples in the community in which they reside. It uses games, discussions, self-reflections, and skill-building exercises to cover the following topics: enhancing relationship quality time, self-esteem and resilience, communication and conflict management, goal setting and implementation, sexual communication and sexual health and reproductive health knowledge, and redefining and challenging norms surrounding IPV occurrence. The formative work guided the protocol, including module duration and timing (2-hour sessions of convenience to participants), ordering of modules (based on potential level of interest and sensitivity of the topics), content (ie, informed scripts of role plays and films), intervention delivery methods (ie, interactive activities), and selection of the interventionists (based on capacity to connect with participants) and venue (community-based, convenient, and safe spaces). *Ghya Bharari Ekatra* was piloted between January and May 2018, and evaluation is presently underway.

**Conclusions:**

Ghya Bharari Ekatra is evidence-based, grounded in intervention-mapping, and developed and iteratively refined using a community-based participatory research approach, suggesting it has great potential to be an acceptable and effective solution to preventing IPV among newly married couples.

**Trial Registration:**

ClinicalTrials.gov NCT03332134; https://clinicaltrials.gov/ct2/show/NCT03332134

## Introduction

### The Impact of Intimate Partner Violence in India

Intimate partner violence (IPV), defined by the World Health Organization as “behavior by an intimate partner or ex-partner that causes physical, sexual or psychological harm, including physical aggression, sexual coercion, psychological abuse and controlling behaviors,” is experienced by approximately one-third (30%) of women worldwide during their lifetime [1]. The National Family Health Survey data suggest that women in India experience IPV with high frequency as well [2], but Indian women of lower socioeconomic status (SES) suffer substantially greater rates, with prevalence estimates ranging from 21% to 99% [3-8]. In addition to being a violation of basic human rights, IPV is associated with numerous negative mental and physical health outcomes and adoption of maladaptive health behaviors [1]. The high IPV prevalence among low-SES populations in India [2,9] and its associated negative health outcomes underscore the need for effective tailored prevention strategies.

### Existing Strategies to Address Intimate Partner Violence

To date, the majority of IPV prevention efforts by the government sector, nongovernmental organizations, and research-based organizations in India has focused on secondary and tertiary IPV prevention [10,11]. However, saturation of legal, mental health, and other IPV support services; high cost and resource limitations hindering expansion of these services; and the high IPV prevalence speak to the need to develop evidence-based affordable, effective, sustainable, and scalable primary prevention strategies to better address the epidemic. Primary prevention in other countries typically begins in schools and colleges and focuses on dating relationships [12]; however, in India, sociocultural barriers, such as social taboos associated with discussing intimacy and sexual relations in schools and colleges [13], and elevated school dropout rates among girls of low SES [14] impede such efforts. The period between engagement and marriage is also not opportune as it is often short and consumed by religious rituals and social and family gatherings for the couple. Fortunately, interventions for primary prevention can be timed later (ie, after marriage) as premarital courtship is still limited (at a nascent stage in the country), 90% of marriages are arranged by parents, and the age of first sexual relationship is often delayed to postmarriage [2,9].

The international literature suggests that most evidence-based IPV prevention interventions engage women alone [10,15,16], men alone [17-21], men and women in parallel gender-concordant groups [22-24], men and women together in large groups [25], or communities (ie, through large-scale community mobilization campaigns) [26,27]. Interdependence theory posits that both intrapersonal and interpersonal dyadic processes serve as determinants of couple’s behavior change [28], suggesting IPV prevention may be most effective, working with the couple as a unit. Our search only found 2 IPV prevention efforts that worked with dyads [29,30], both of which demonstrated reductions in IPV but were developed and conducted in resource-rich settings. In India, 3 major evidence-based interventions have been developed or adapted and tested but again worked solely with women [10], men [19], or mother-in-law and daughter-in-law pairs [31]. We herein describe the development of a dyadic intervention for the primary prevention of IPV among newly married couples residing in slum communities. Specifically, we provide the evidence and theoretical basis for the intervention content and delivery.

## Methods

### Study Overview and Use of the Intervention Mapping Framework

The intervention, *Ghya Bharari Ekatra* (*take a flight together*), was developed using a community-based, mixed-methods design, rooted in couple-interdependence theory and guided by the intervention mapping (IM) framework outlined by Bartholomew et al [32]. IM is a systematic approach to intervention planning, implementation, and evaluation that is driven by evidence, theory, and community participation [32]. It involves 6 critical steps: (1) a needs assessment, (2) preparation of matrices of change objectives, (3) selection of theory-based methods and practical applications, (4) production of intervention components and materials, (5) intervention adoption and implementation, and (6) evaluation planning.

The methods used to develop the intervention include (1) cross-sectional surveys with newly married men and women residing in slums to identify correlates of IPV to inform intervention change objectives, (2) 21 key informant interviews (with individuals who bring expertise in IPV, gender equality, marital health, sexual and reproductive health, or work with slum communities) to inform the content and delivery of the intervention, (3) feedback from gender-based violence (GBV) experts and the Indian Council of Medical Research-National AIDS Research Institute (ICMR-NARI, Pune, India) community advisory board (CAB) on the intervention protocol, and (4) 3 focus group discussions with married men, married women, and parents-in-law (each of 7-10 participants) to assess acceptance of some of the more controversial topics included in the intervention.

The intervention was developed in Pune, India, the second largest city in the western state of Maharashtra. It has a population of 3.1 million, a female:male sex ratio of 0.948, and significant religious diversity (79% [2,449,000/3,100,000] Hindu; 11% [341,000/3,100,000] Muslim; 4% [124,000/3,100,000] Buddhist; 2% [62,000/3,100,000] Jain; and 2% [62,000/3,100,000] Christian). Approximately, a quarter (22% or 690,545 individuals) of Pune resides in slums [33].

### Trial Registration, Ethics, Consent, and Institutional Board Approval

The study was approved by the Ethics Committee of ICMR-NARI and the institutional review board of Emory University (Atlanta, USA) and registered at ClinicalTrials.gov (NCT03332134) and the Clinical Trials Registry-India (CTRI/2018/01/011596).

### Intervention Mapping Step 1: Needs Assessment

First, we conducted a systematic review of the literature to assess the breadth and depth of IPV in India and to identify high-risk groups (ie, women of low SES) [34]. Confirmation of IPV as a major priority area for the target community came from prior work within the Pune community [9,35], regular reporting on IPV by local media [36,37], and recognition of need for the development and evaluation of an evidence-based IPV intervention by ICMR-NARI CAB.

**Figure 1:**
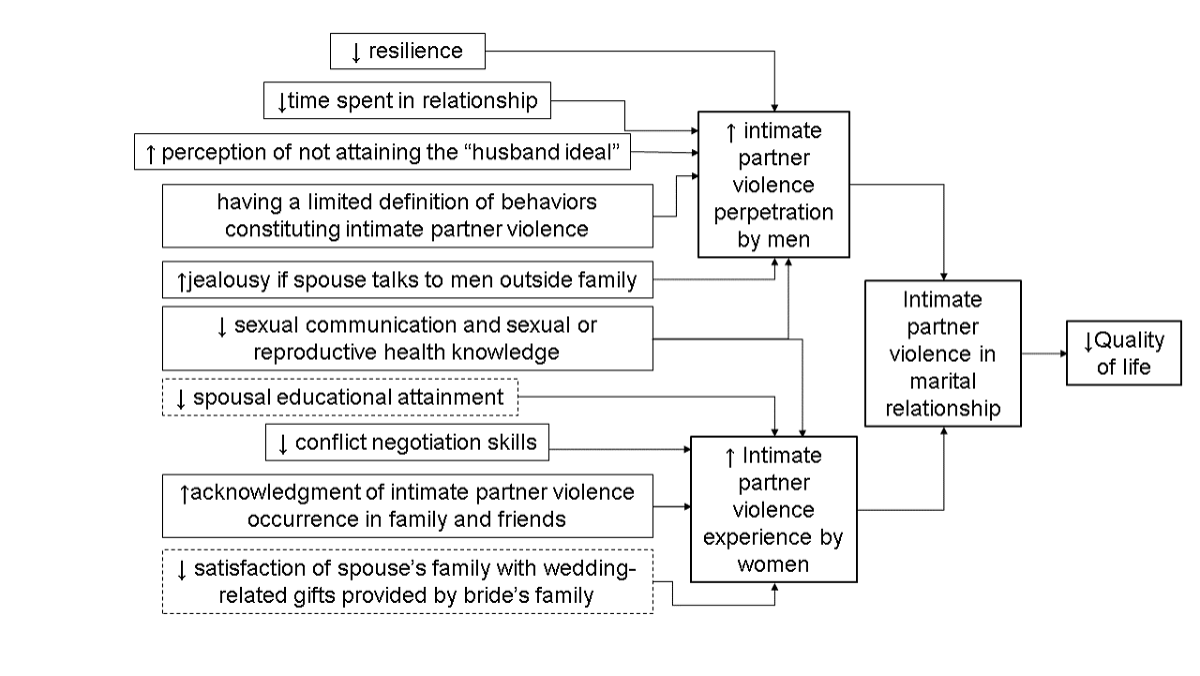
A logic model of the needs assessment.

Afterward, an analysis of IPV causation in Pune slums was undertaken to inform the development of the PRECEDE logic model (Figure 1). This included identification of correlates of IPV perpetration and experience through respective cross-sectional surveys with 100 newly married male and 100 newly married female residents of Pune slums and extraction of themes regarding IPV causation from key informant interviews.

We then assessed the community capacity by exploring services provided by local community-based organizations (CBOs); engaging in informal discussions with community leaders and individuals working in the intervention communities (ie, from *anganwadis* [Integrated Child Development Scheme, ICDS, child care centers] and *mitramandals* [male youth social clubs]); reviewing Indian laws, policies, and government schemes that address IPV; and surveying the physical environment (ie, space to conduct the intervention) and local resources (ie, police stations for safety concerns).

### Intervention Mapping Step 2: Preparation of Matrices of Change Objectives

To develop matrices of change objectives (Multimedia Appendix 1, we used the IPV correlates noted in IM step 1 to draw causal pathways for influencing change in IPV perpetration for men and IPV experience for women, defined the desired behavioral outcomes of the intervention, and then subdivided the behavioral outcomes into performance objectives. Afterward, the stated performance objectives were linked with key, changeable determinants informed by behavior change theories [32] to ultimately define and prioritize the change objectives for the intervention.

### Intervention Mapping Step 3: Selection of Theory-Based Methods and Practical Applications

Together with the research field team (that brought working knowledge of the needs and interests of individuals residing in Pune slums through years of field experience), we brainstormed ideas for the intervention that would accomplish each of the change objectives. We narrowed the list of applications based on the extent to which we felt they could appeal to and meet the needs of the intervention population and whether the application was justified by a theory-based behavior change method. The selected intervention applications were individually again examined by ASK, SS, and RS to ensure they addressed the respective change objectives.

### Intervention Mapping Step 4: Production of Intervention Components and Materials

The phase II key informant interview data were used to inform the production of the application content and materials. Specifically, we extracted case stories and common marital conflict scenarios, and key informant suggestions about information content, delivery methods (including language considerations), order of module delivery, and duration. We then reviewed and revised the content and delivery for the 6-intervention modules exercise-by-exercise until team consensus was achieved about the material being relevant, stimulating, clear, and appropriate in addressing the change objectives.

Thereafter, the intervention materials and delivery strategy were presented to the ICMR-NARI CAB and regional GBV experts and revised based on their feedback. The content of specific exercises for which there remained uncertainty about participant acceptance or comprehension (ie, exercises related to sexual communication and knowledge, career planning, and government schemes) was presented to the community for feedback through 3 focus group discussions with married men (n=7), married women (n=7), and mothers-in-law and fathers-in-law (n=8). Finally, the research team worked intimately with the module publishers and film producers (for the short films used in module 3 to ensure quality and appropriateness of the final product.

### Intervention Mapping Step 5: Intervention Adoption and Implementation

We brainstormed potential adopters of the intervention, consulting recommendations from the qualitative data about existing government and nongovernment programs with whom to partner to enhance future scalability and sustainability of the intervention. The brainstorming included free listing of community gatekeepers who could raise awareness about the intervention and recruit participants, individuals and agencies who could deliver the intervention, and potential intervention venues. Subsequently, we met the individuals and agencies to gauge their interest, capacity, and processes they required to formally establish the partnership. Selected interventionists underwent a 1-week interactive training. Necessary paperwork for partnering agencies was completed.

### Intervention Mapping Step 6: Evaluation Planning

To develop the plan for evaluation of intervention effect, we consulted the matrices of change objectives and the scientific literature to find appropriate, validated tools to measure the desired change. In developing the measures of acceptance, feasibility, and safety, we ensured the evaluation included the perspectives of multilevel stakeholders (ie, participants, interventionists, police stations, CBOs, and community members) as is emphasized by Bartholmew et al [32]. Process indicators related to fidelity, dose delivered, dose received, reach, recruitment, and context were developed in consultation with the Consolidated Standards of Reporting Trials guidance for pilot trials [38] and the process evaluation guide by Saunders et al [39].

## Results

### Summary

**Figure 2:**
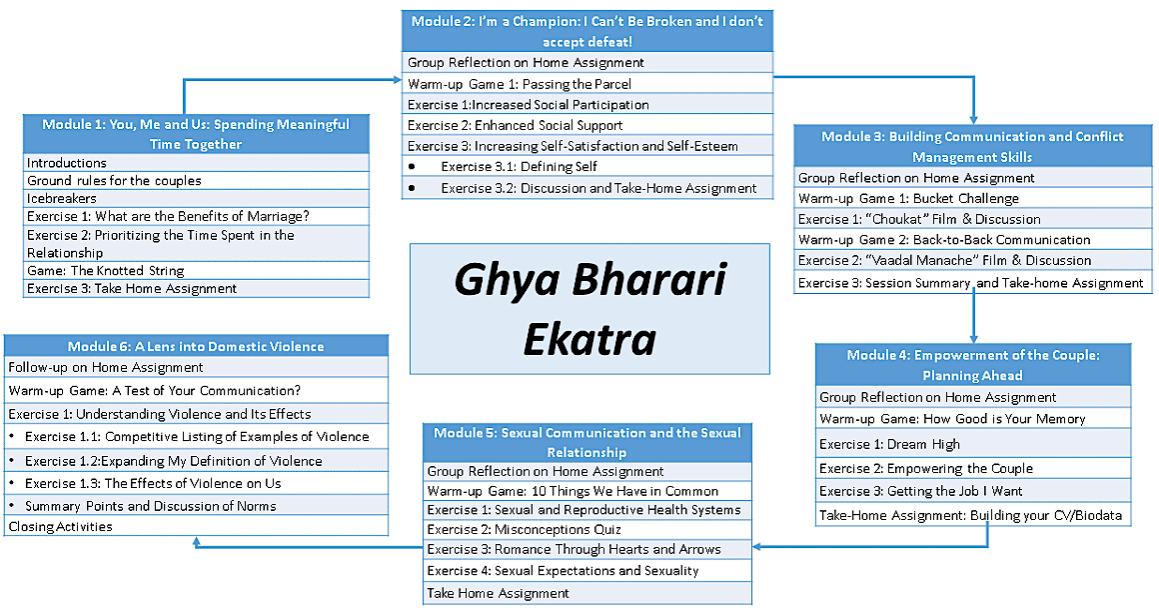
The *Ghya Bharari Ekatra* intervention.

The resulting *Ghya Bharari Ekatra* intervention (Figure 2) was a 6-session intervention that was intended to be delivered by a trained pair of male and female community educators to groups of 3 to 5 newly married couples in the slum community in which the participants resided. Community educators were lay people who either belonged to the community in which the intervention was conducted or were members of other communities interested in grassroots-level community work. The intervention employed competitive games, intensive discussions, self-reflections, and skill-building exercises and covered the following topics in 2-hour sessions over 6 weeks: enhancing relationship quality time, self-esteem and resilience, communication and conflict management, goal setting and implementation, sexual communication and sexual health and reproductive health knowledge, and redefining and challenging norms surrounding IPV occurrence. All sessions were delivered to groups of couples with the exception of the sexual communication and sexual and reproductive health module, which was to be delivered in gender-concordant groups of 3 to 5. In this section, we demonstrate how the results of each IM step contributed to the development of the intervention.

### Intervention Mapping Step 1: Needs Assessment

The systematic review [34], formative work [9,35], and NARI CAB meetings identified the need for a targeted primary IPV prevention intervention for low-income, slum communities in India. The cross-sectional surveys and key informant interviews isolated key behavioral factors contributing to IPV in this community and led to the development of the PRECEDE logic model (Figure 1). The assessment of community capacity highlighted that individual slum communities had many resources (ie, venues) to foster implementation of the intervention: community halls, *anganwadis*, *mitramandals*, *mahilabachatghat* groups (women’s savings groups), CBOs, and schools.

### Intervention Mapping Step 2: Preparing the Matrix of Change Objectives

Figure 1 was used to define and prioritize, based on changeability and importance, the desired behavioral outcomes for each of the 6 intervention modules. Each of the behavioral outcomes became the focus of a session in the 6-session intervention: (1) increased quality time spent in the relationship, (2) increased self-esteem and resilience, (3) enhanced communication and conflict management skills, (4) improved goal setting and goal implementation skills, (5) improved sexual communication and sexual health and reproductive health knowledge, and (6) expansion of definitions of behaviors constituting IPV and challenge subjective norms surrounding IPV occurrence. For each of the 6 sessions, individual matrices of change objectives were developed (Multimedia Appendix 1).

### Intervention Mapping Step 3: Selection of Theory-Informed Intervention Methods and Practical Applications

Couple’s interdependence theory is the unifying theory in which the intervention is grounded [28]. The Interdependence model [28], which we used to guide intervention development, has 3 major components: (1) *transformation of motivation* or shift in orientation from self- to relationship-centered (ie, the husband internalizing the impact of IPV and poor relationship quality on his spouse and the relationship), (2) *communal coping* in which the dyad has a shared assessment of the threat of IPV and poor relationship quality to their health and quality of life and share a vision of the action plan to reduce IPV and improve relationship quality, and (3) *behavior change* through which the couple together adopts and sustains behaviors to reduce IPV and enhance relationship quality. In addition to couple’s interdependence theory, several theoretical behavior change methods along with the change objectives guided the development of the practical applications for each module (Multimedia Appendix 2).

### Intervention Mapping Step 4: Development of Intervention Components and Materials

The qualitative data guided the intervention duration, module order, content, and delivery.

#### Module Duration and Order

Key informant interviews provided guidance on module duration and order of delivery. Participants placed emphasis on the need for intervention timing to be convenient for participants, for interventionists to be respectful of the participant’s time, and for sessions to be limited to 2 hours. An HIV researcher with expertise in working with slum communities guided:

Many will say “our time is up, we are leaving”…You can give training for 1 hour, at the most 2 hours...Whatever time we tell [them], we should do within that.

Although key informants had varying suggestions for ordering of modules, most agreed on beginning with relationship building, as it was the foundation of married life. A founder of a marital counseling CBO expanded on the importance of prioritizing this module:

Because many times they [participants] don’t know...I mean, understanding wife, or understanding husband, usually as marriages are always between the families...they have seen [their respective partner] maybe at the time of marriage or maybe only at the event of meeting the girl, otherwise they don’t know what is [a marital relationship]...And then...first 3-4 months, especially in our communities, there are so many festivals and this and that and go to this temple, that temple, and then there is no time to [spend with each other], you know?

Other common themes about module order included the need for initial intervention sessions to have particularly high appeal (ie, provide information about relevant government services and economic empowerment), delaying the sexual and reproductive health session until group rapport and comfort were established, and ensuring each module built on information provided in the prior sessions. Finally, 1 key informant who works with a CBO that focuses on women’s empowerment and gender equality through male engagement highlighted the importance for the session about domestic violence (DV) to occur at the end to minimize attrition:

And anyways, when we talk of domestic violence to begin with. Then men think, “these people have this only.” [That this is the only agenda this organization is coming to us with]. They get put off by that. We should take that [the issue of violence] up last.

#### Intervention Delivery

To engage and retain participants, key informants emphasized the need for the intervention to be fun, interactive, and activity-based and to make use of audio-visual tools:

If you just give lectures, nobody will come to you.

Gynecologist

The more audio-visual films you will use, the more impact you will have.

DV lawyer

You need to make it as engaging and as participatory as possible.

GBV researcher

Specific delivery methods (ie, competitions, games, role plays, self-reflection, practice with feedback, songs and dance therapy, and quizzes) and audio-visual tools (ie, flip charts, anatomic models, and films) suggested by the key informants were incorporated throughout all 6 sessions. To foster couple interest and engagement in the sessions, a *star points competition* was woven throughout the 6 sessions, wherein couples accumulated points for participation, punctuality, and the games they won, and ultimately, the couple with the most points at the final session was recognized and received an award.

Key informant data also led us to repeatedly evaluate the language used in the modules to ensure it was easy to understand, familiar to the participants, and also scientific. For example, a social worker of a CBO that focused on women’s empowerment and gender equality through male engagement advised:

Many times to make it palatable for the slum area people, we use many times the words, which are, they use for their body parts. Now, to begin with this is good, but ultimately we should bring them to the real scientific terms of that body part.

Key informants also provided guidance on which exercises should be performed individually, in couples, and in groups. For example, the input from a counselor of a marital counseling CBO led to the sexual communication and sexual and reproductive health session being delivered in separate gender-concordant groups:

First, sexuality you should conduct in a group and separately too. Because, if the participant has to speak openly, then [conducting men and women group sessions], limitations will come for that. Even if you take a couple...if they are husband and wife, even then, to talk to someone else in front of my partner will be difficult for me.

#### Content

Individual module content was also informed by key informant data. For example, in response to the advice provided by the director of a CBO that engaged men to promote gender equality below, we designed an activity in module 1 in which couples self-reflected about the time they dedicated to their relationship and strategies to increase that time:

This is a relationship program and you can’t talk about relationships without emotions and without reflections and without communications…You actually need to bring in the self-reflection.

As part of the second and fourth sessions, which respectively aimed to build resilience and empower the couple, interactive informational sessions were held with community resource people (ie, social workers, government scheme enrollment officers, and *bhishi group* leaders) to provide relevant information about community resources. These sessions were derived from key informants stressing the need for the intervention to be linked to existing structures and tailored to the needs of and services available in individual slum communities:

The needs of one community may not be the needs of the next [community]. So it will also be important to cater it in that sense. As in, “this is our module, and we now have to use this,”—we can’t do that.

DV lawyer

She further expanded that:

...connecting them to government systems becomes important so that it’s longstanding.

The scripts for the module 3 films, used to provoke discussion about effective communication and conflict management strategies, were based on conflict scenarios taken directly from examples provided by our key informants and observations made by our field team. While in the field, our team noted that conflict in the newly married couples often arose from differences in expectations resulting from the newly wed women having moved to the urban slums from surrounding rural areas following marriage, whereas the men had long resided in urban slum environments. This was substantiated by key informant examples. For example, an ICDS project officer, responsible for overseeing *anganwadi* workers explained:

Soon after marriage, one girl came...and she was from a rural area. At that time, when that girl came into the slum, and at that time, the boy was from Pune, so accordingly he had many expectations, that “my wife should wear jeans.”...But she wasn’t used to jeans, because she was from a typical, rural area. “So then how can I do it?” About that they both started arguing.

Module 5 content was derived from the topics identified by key informants as critical to cover (ie, reproductive health systems, conception and pregnancy, communicating sexual expectations, and creating romance). Items for the module 5 misconceptions quiz were pulled directly from misconceptions reported by key informants (ie, penile length being associated with pleasure, pornography guiding performance of sexual intercourse, and erectile drugs being misused). A marital counselor provided the following:

Most of [the men] do sexual abuse in accordance with what they watch in blue films and they perform similar acts on their wives leading to harassment and unnatural sexual acts. This attitude can be changed through proper education.

We added content about sexuality and creating romance based on a suggestion by a key informant who directed a gender-equality CBO that engaged men:

How physical relationships are connected to emotional bonding, we need to focus on this too. Otherwise, if we look at it (marital sexual relationship) only from the perspective of bodily need, then to strengthen the bond in this relationship becomes very difficult.

Finally, module 6 content was developed in response to key informants emphasizing the need to challenge deep-rooted norms of DV acceptance and to enable participants to expand their definitions of behaviors constituting abuse (taking into account the survivor perspective). A feminist sociologist and a DV lawyer informant, respectively, suggested the means for doing so:

What men perceive as violence and what women perceive as violence is very different. I think we need to tease that out.

Feminist sociologist

To be able to say that you know violence is subjective and that we’ll have to understand it from the victim’s perspective.

DV lawyer informant

The finalization of the intervention name, *Ghya Bharari Ekatra*, involved research team members, in consultation with the community, brainstorming and short listing titles that were memorable, appealing, and best depicted a couple’s joint empowerment. Searches of other Indian empowerment programs and interventions were conducted to ensure uniqueness. The title was ultimately determined by vote among research team members.

### Intervention Mapping Step 5: Planning for Intervention Adoption, Implementation, and Sustainability

The decision to engage male and female community educators as the primary interventionists, to bring in community resource people to lead specific intervention exercises, to work with *anganwadi* workers in recruiting participants, and to utilize community-based venues was driven by key informant data.

#### Interventionists

In selecting interventionists, key informants stressed the need to ensure that the candidate was interested in the intervention, had strong oratory and critical thinking skills and gender-equitable attitudes, capacity to make the participants feel safe and secure, and sensitivity (to note when a participant seemed uncomfortable). Many highlighted the need for the interventionists to be of a similar demographic to the participants to best connect with them. For example, a counselor from the marital counseling CBO described who an ideal interventionist would be:

[People from] the slum community and the ordinary citizens of the society...those who have led successful lives…those who have brought up kids. Those who think that their lives have been spent happily, you should involve such people, because they are a role model in front of them.

The director of a CBO that engaged men to promote gender equality further emphasized this:

...to whom do people connect? To those whose language they are accustomed, to them they connect.

He also elaborated on the need for the interventionists to be emotionally engaged with the participants’ community:

If you are not part of people’s life, people are not part of your project, OK? In that community, in that couple, someone in that couple’s mothers has died during the period [of the intervention], someone’s father has died. It’s someone’s 10th[10thday death memorial ceremony]...If your intervention team isn’t part of their lives, they [the participants] aren’t part of our procedures emotionally.

Key informants emphasized the importance of some sessions to be delivered by content-level experts (ie, legal, health, and social workers and mental health counselors), community resource people (ie, *bachatghat* group leaders and community welfare officers), and engaging *anganwadi* workers to help with recruitment and retention, given their existing rapport with community members and likely interest in the intervention. Two marital counselors separately emphasized the need for some content to be delivered by gender-concordant facilitators:

However much you might think that they [the participants] will not feel shy, it is not at all like that. Hence while conducting the husband’s sex education, you keep a gents community worker [have a male community worker as the facilitator]...and while conducting for ladies, you keep a lady [have a female community worker as the facilitator].

When a woman tells a man, it remains ineffective but when a man tells another man, about what is DV, and how it is contracted and how should one live in day-to-day lives, then the attitude will change. The attitude of looking at domestic violence should change.

Thus, we ultimately decided to have the sessions facilitated by a male and female community educator, who demonstrated strong oratory, group facilitation, critical thinking skills, and community involvement during the behavioral interviewing process. Content-level experts (ie, medical officers) and community resource people (ie, social workers and *bachatghat* leaders) were also brought in for the second, fourth, and fifth session, and participants were provided contact information for other services that provided support for IPV, substance abuse, and legal, medical, and mental health. Session 5 was the only session delivered in gender-concordant groups (as opposed to groups of couples) and was delivered by gender-concordant doctors. In addition, the research team met the local ICDS administration to obtain permission for *anganwadi* workers to help facilitate recruitment and retention efforts.

#### Training

A 6-day training for the community educators was held at ICMR-NARI by the research team. It was conducted in Marathi and Hindi and covered basic research ethics and safety, DV, community educator responsibilities (including need to report DV and other safety concerns to the study team), and the content of each of the 6 intervention sessions. First, research team members role-played the delivery of each session. Afterward, community educators mock delivered the session with feedback from the research team and other community educators.

#### Venue

Key informants highlighted the need for the venue to be of choice and convenience to the participants, in a setting where privacy could be maintained, a safe space where participants would feel comfortable sharing, and where discipline could be maintained. A trainer from a CBO, who promoted gender equity and justice, advised that for participants to be engaged, the research team had to dedicate effort to creating a safe space:

People would talk to you, if you actually create that space, very safe for sharing—not just between the trainer and trainees, but also amongst the group of participants, right? How safe they would feel in a physical structure, in a room, in a hall...How safe they feel with the gadgets around, mobile phones, audio-recorders, or video camera...You know, it all depends on the way you create that environment.

Although health care settings were mentioned as optimal spaces for maintaining privacy, they carried associated stigma and fear of infectivity, cost, and challenges of distance and insufficient space. *Outings* (ie, gardens and trips) were also suggested because they could serve as fun, exciting lures for potential participants, and potentially ensure the couples’ presence for the entirety of the session, but concerns were raised about time in travel and whether participants would be permitted to leave their community (particularly, if pregnant). Many key informants suggested having the venue in the slum community itself out of convenience, accessibility, and ease of obtaining family member’s permission for the couple to attend, but noted that we may encounter difficulty maintaining privacy and finding space in such venues. Suggested community-based venues included *anganwadis*, schools, community halls, and religious venues. Religious venues brought the advantage of being acceptable but were often cited as lacking privacy, having sociopolitical affiliation, challenging secularism, and having limited availability (ie, serving as child care centers by day).

Factoring in the concerns raised by the key informants, we ultimately decided to hold the intervention in the slum community from which the participants were drawn. Venues were selected in partnership with community educators, weighing likelihood of safety and privacy. In addition, community educators and field team members were provided scripts and protocols for handling specific violations of privacy and safety. Finally, community meetings were held before the intervention delivery to help avert community misconceptions and undue stigma associated with the program.

### Intervention Mapping Step 6: Planning for Evaluation

The detailed evaluation plan, developed using the Multimedia Appendix 1 matrices of change, is presented in Table 1. It includes both outcome and process indicators and assesses preliminary efficacy, safety, feasibility, and acceptance. Evaluation methods include a preintervention and 3-month postintervention survey administered to participants, postsession open-ended discussions with participants to assess acceptance, postsession open-ended discussions with community educators to assess acceptance and challenges with delivery, research team logging of recruitment and retention numbers and associated facilitators and barriers, and semistructured evaluation of fidelity, dose delivered, and dose received by the research team during each session.

**Table 1:** Intervention evaluation plan.

Indicator and assessment method			Data source
**Outcome indicators**			
	**Participants spend more quality time together in the relationship**		
		Time spent in relationship	M^a^ (S^b^)
		Satisfaction with time spent in relationship	M (S)
	**Participants experience enhanced self-esteem and resilience**		
		Resilience (CD-RISC-10^c^)	M (S)
		Extent to which feel attained qualities of *ideal husband*	M (S)
		Jealousy if spouse talks to other men	M (S)
		Self-esteem (RSES^d^)	M (S)
	**Participants develop enhanced communication and conflict management skills**		
		Conflict negotiation skills (CTS2N^e^)	F^f^ (S)
		Confidence in communicating various scenarios with partner	M/F (S)
	**Participants develop enhanced confidence in goal setting and goal-implementation skills**		
		Extent to which feel attained qualities of *ideal husband*	M (S)
		Confidence in setting and achieving goals, and listing resources that support achieving goals	M (S)
	**Participants develop enhanced sexual communication and sexual and reproductive health knowledge**		
		Confidence in sexual communication	M/F (S)
		Reproductive health beliefs	M/F (S)
	**Participants’ definitions of behaviors constituting IPV^g^** **will expand and will be less accepting of IPV**		
		Definition of DV^h^ (using items from abridged IFVCS^i^)	M (S)
		Attitudes toward DV acceptance (ATWBS^j^)	F (S)
	**Overall reduced DV**		
		Past 1-month DV experience (abridged IFVCS)	F (S)
**Process indicators**			
	**Fidelity**		
		Extent to which training provided as planned	RT^k^
		Extent to which interventionists delivered each activity as intended
		Difficulty interventionists experienced in delivering intervention content	PE^l^
	**Dose delivered**		
		Number of sessions delivered by PE	RT
		Extent to which expected content delivered	RT
		Extent to which intended intervention material used	RT
		Time required to deliver each activity	RT
	**Dose received (exposure and satisfaction)**		
		Participant engagement in each session activity	RT
		Participant completion of home assignments	RT
		Participant understanding of content of each activity	M/F
		Participant satisfaction with each session, timing, and duration of intervention	M/F
		Family satisfaction with subject’s participation in intervention	M/F
		Participant satisfaction with interventionists	M/F
		Participant acceptance of safety measures	F (S)
		PE satisfaction with each session	PE
	**Participant recruitment and retention**		
		Number approached and number of attempts to reach potential participant pre-enrollment	RT, M/F
		Time between initial contact with community gatekeeper and completion of recruitment	RT, M/F
		Family presence during permission process	RT, M/F
		Number consented and barriers to consenting	RT, M/F
		Number assessed for eligibility, and ineligibility reasons	RT, M/F
		Number randomized	RT, M/F
		Number of barriers to baseline and 3-month survey completion; staff attempts and reminders	RT, M/F
	**Reach or participation rate**		
		Number or duration of sessions participant attended and barriers to attendance	RT, M/F
		Number terminated, exited from the study and reason	RT, M/F
		Number of staff reminders and home visit reminders	RT, M/F
		Number of staff calls or home visits for missed sessions	RT, M/F
	**Context**		
		Response from police, community leaders, and family members when household approached	RT
		Number of community sensitization meetings held, number in attendance, and response to or acceptance of intervention	RT
		Barriers or facilitators encountered by the study team in implementing the program	RT
	**Safety**		
		How research is being discussed in the community	PE
		1-month DV experience	PE; RT; F (S)
		Family conflict attributed to intervention	PE; RT; F (S)
		DV reported by participants (or noted by staff) at Women’s day or during intervention	PE; RT; F (S)
		Adverse events	PE; RT; F (S)
	**Additional feasibility indicators**		
		Number of potential PE approached and eligible to serve as interventionists and associated barriers	RT
		Number of PE completion of training	RT
		Number of community key persons, E-seva Kendra officials, and medical officers attending sessions 2, 4, and 5, respectively (and associated barriers)	RT
		Venue identification, privacy, and retention	RT
		Cost of intervention delivery	RT

^a^M: male participant.

^b^S: pre- and postintervention survey item.

^c^CD-RISC-10: Connor-Davidson Resilience Scale-10 item.

^d^RSES: Rosenberg Self-Esteem Scale.

^e^CTS2N: Conflict Tactics Scale-2 Negotiation Subscale.

^f^F: female participant.

^g^IPV: intimate partner violence.

^h^DV: domestic violence.

^i^IFVCS: Indian Family Violence and Control Scale

^j^ATWBS: Attitudes Toward Wife Beating Scale.

^k^RT: research team.

^l^PE: peer educator.

## Discussion

### Principal Findings

Primary prevention of IPV in low-SES communities in India is critical, as IPV is not only a human rights violation but also a critical public health problem, with high frequency and significant associated psychosocial and physical morbidity. *Ghya Bharari Ekatra* is evidence-based, developed using theory (ie, couple-interdependence theory as a unifying framework as well as individual behavior change theories to inform intervention methods), grounded in intervention-mapping, and developed and iteratively refined using a community-based participatory research approach, suggesting it has great potential to be an acceptable and effective solution to preventing IPV among newly married couples. The greatest strengths of *Ghya Bharari Ekatra* include that it is peer-led, community-based, interactive, and introduces novel concepts that are of importance to the participants. Furthermore, integration of the intervention into existing community resources and government program infrastructure will foster future sustainability and scalability throughout slum communities in India if it is deemed effective.

### Pilot Study Progress

Enrollment into the pilot study to assess the safety, acceptance, feasibility, and preliminary efficacy in 40 couples (20 intervention and 20 control) commenced in January 2018 and was completed in May 2018. Preliminary feedback from participants, community educators, and the research field team suggests the intervention was highly accepted and safe. Three-month follow-up visits have been completed, and pilot results will be available in the spring of 2019. If pilot results are promising, the efficacy of *Ghya Bharari Ekatra* will be tested on a large scale throughout India.

## Appendix

Multimedia Appendix 1 Matrix of change objectives.

Multimedia Appendix 2 Theory-informed methods and practical applications.

## Abbreviations

ATWBS: Attitudes Toward Wife Beating Scale
CAB: community advisory board
CBO: community-based organization
CD-RISC-10: Connor-Davidson Resilience Scale-10 item
CTS2N: Conflict Tactics Scale-2 Negotiation Subscale
DV: domestic violence
F: female participant
GBV: gender-based violence
ICDS: Integrated Child Development Scheme
ICMR-NARI: Indian Council of Medical Research-National AIDS Research Institute
IFVCS: Indian Family Violence and Control Scale
M: male participant
PE: peer educator
IM: intervention mapping
IPV: intimate partner violence
RSES: Rosenberg Self-Esteem Scale
RT: research team
S: pre- and postintervention survey item
SES: socioeconomic status
