# Supplementary material for: Primary Prevention of Intimate Partner Violence Among Recently Married Dyads Residing in the Slums of Pune, India: Development and Rationale for a Dyadic Intervention
Source: JMIR Res Protoc. 2019 Jan 18;8(1):e11533. doi: 10.2196/11533 (PMC6356185; doi:10.2196/11533)
Supplement: Multimedia Appendix 1 [file resprot_v8i1e11533_app1.pdf]

<table>
<tr><td></td><td colspan="3">Personal Determinants</td></tr>
<tr><td>Performance Objectives</td><td>Understanding</td><td>Changing habitual behavior</td><td>Evaluation</td></tr>
<tr><td rowspan="2"><u>Module 1</u>: Participants spend more quality time together in the relationship.</td><td>Recognize the importance of marriage and partnership.</td><td>Analyze existing barriers to spending meaningful time with partner.</td><td rowspan="2">Assess success in carrying out the plan to challenge existing barriers to spending meaningful time together.</td></tr>
<tr><td>Recognize the benefits of spending meaningful time together.</td><td>Demonstrate design of a plan to challenge existing barriers to spending meaningful time together.</td></tr>
<tr><td></td><td>Understanding</td><td>Knowledge</td><td>Self-efficacy</td></tr>
<tr><td rowspan="3"><u>Module 2</u>: Participants experience enhanced self-esteem and resilience.</td><td>Recognize self and self-worth.</td><td>List strategies that help build self-esteem</td><td>Practice strategies that help build self-esteem.</td></tr>
<tr><td rowspan="2">Discuss expectations that are and are not feasible to meet.</td><td>Identify positive versus negative coping mechanisms and their effects.</td><td rowspan="2">Schedule activities that increase social participation.</td></tr>
<tr><td>List and recognize existing support systems.</td></tr>
<tr><td></td><td>Understanding</td><td>Self-efficacy</td><td>Evaluation</td></tr>
<tr><td rowspan="3"><u>Module 3</u>: Participants develop enhanced communication and conflict management skills.</td><td>Identify negative versus positive conflict management and communication.</td><td rowspan="3">Practice effective communication skills.</td><td rowspan="3">Assess success in practicing positive communication and conflict resolution methods.</td></tr>
<tr><td>Summarize the effects of negative versus positive conflict management and communication.</td></tr>
<tr><td>Identify communication barriers and means for overcoming them.</td></tr>
<tr><td></td><td>Understanding</td><td>Knowledge</td><td>Self-efficacy</td></tr>
<tr><td rowspan="3"><u>Module 4</u>: Participants develop enhanced confidence in goal-setting and goal-implementation skills.</td><td rowspan="3">Describe benefits of empowerment.</td><td>List available community services that support goal planning.</td><td>Set achievable goals.</td></tr>
<tr><td>Describe process necessary to use government subsidies, schemes, and other resources.</td><td>Practice goal planning</td></tr>
<tr><td>Recognize positive versus negative interviewing methods.</td><td>Practice developing a Curriculum Vitae (CV).</td></tr>
</table>

	Understanding	Knowledge	Self-Efficacy
	Describe what constitutes a good sexual partner.	List means of increasing romance.	Practice effective sexual communication
Module 5: Participants develop enhanced sexual communication and sexual and reproductive health knowledge.	Explain the process of menstruation and conception.	Name the parts and functions of the male and female sexual and reproductive health systems.	
		Identify misconceptions about reproductive health issues that often result in domestic violence.	
		List pregnancy support services.	
	Discuss the importance of understanding partner's sexual expectations and concerns.	Identify misconceptions about sex and sexuality.	
	**Understanding**	**Knowledge**	**Evaluation**
Module 6: Participants' definitions of behaviors constituting IPV will expand and will be less accepting of IPV	Describe the effects of DV on the survivor, perpetrator, family, and children.	Define comprehensively behaviors constituting DV.	Critique the belief that violence is situationally acceptable and useful.

Table 1. Matrix of Change Objectives
