# Supplementary material for: Primary Prevention of Intimate Partner Violence Among Recently Married Dyads Residing in the Slums of Pune, India: Development and Rationale for a Dyadic Intervention
Source: JMIR Res Protoc. 2019 Jan 18;8(1):e11533. doi: 10.2196/11533 (PMC6356185; doi:10.2196/11533)
Supplement: Multimedia Appendix 2 [file resprot_v8i1e11533_app2.pdf]

**<u>Module 1 PO</u>: Participants spend more quality time together in the relationship.**		
**Change objectives**	**Methods**	**Applications**
Recognize the importance of marriage and partnership.	Persuasive communication; Discussion	1. Participants group-reflect on the potential benefits of a life partner by assessing life partnership through a lens of various other relationships they are more familiar with (i.e. business partner, family, friends)
Recognize the benefits of spending meaningful time together.	Framing	1. Participants individually specify the time spent together on a typical day<br>2. The group reflects on the time spent (i.e. sufficiency, quality), importance of spending time, and barriers to doing so<br>3. Couples develop a practical plan to increase quality time spent together
Describe existing barriers to spending meaningful time with partner.	Planning coping response	
Demonstrate design of a plan to challenge existing barriers to spending meaningful time together.		
Assess success in carrying out the plan to challenge existing barriers to spending meaningful time together.	Guided practice	1. Home assignment to practice implementing a strategy they have identified to spend more time together<br>2. Group reflection about how effective they were in implementing the strategy (beginning of Session 2)
**<u>Module 2 PO</u>: Participants experience enhanced self-esteem and resilience.**		
**Change Objectives**	**Methods**	**Applications**
Schedule activities that increase social participation.	Goal setting	1. Participants informed of upcoming social activities in their community and asked about which they plan to attend and what other activities they would like to see offered to foster interaction.
List and recognize existing support systems.	Individualization	1. Participants individually reflect on existing support system for common stressing scenarios<br>2. Group diagrams positive and negative coping responses to a stressing scenario (i.e. job loss) and uses the diagram to reflect on the consequences of each of the responses<br>3. Community informant Q&A session regarding available community support services
Identify positive versus negative coping mechanisms and their effects.	Discussion	
Recognize self and self-worth.	Self-affirmation task	1. Within couple exercise where individuals tell their partner about how they see themselves (origins, interests, strengths, easy and difficult-to-change weaknesses)<br>2. Game in which individual is to guess the characteristic their partner appreciates most in them
Discuss expectations that are and are not feasible to meet.		
List strategies that help build self-esteem	Discussion	1. Group develops a list of strategies that can be employed to boost self-esteem

Practice strategies that help build self-esteem.	Guided practice	1. Individuals choose a strategy from the list to practice at home the following week.<br>2. Group reflection about how effective they were in implementing the strategy (beginning of Session 3)
**<u>Module 3 PO</u>: Participants develop enhanced communication and conflict management skills.**		
**Change Objectives**	**Methods**	**Applications**
Identify negative versus positive conflict management and communication.	Scenario-based risk information	1. Participants view two short films of common conflicts couples encounter post-marriage<br>2. Films are used to guide discussion regarding positive and negative communication and conflict management methods, the associated effects, and barriers to effective communication and conflict management as well as strategies for overcoming them
Summarize the effects of negative versus positive conflict management and communication.	Scenario-based risk information	
Identify communication barriers and means for overcoming them.	Planning coping responses	
Practice effective communication skills.	Guided practice	1. Participants practice effective communication skills while discussing future goals with partner in home exercise<br>2. Group reflection about the extent to which effective communication skills were employed in discussing differences in goals with partner (beginning of Session 4)
Assess success in practicing positive communication and conflict resolution methods.		
**<u>Module 4 PO</u>: Participants develop enhanced confidence in goal-setting and goal-implementation skills.**		
**Change Objectives**	**Methods**	**Applications**
Set achievable goals.	Goal setting	1. Couples define and specify achievable 5-year goals
Practice goal planning.	Goal setting	1. Couples develop stepwise plans for achieving goals. Facilitators provide feedback and suggest community resources to help support achievement of goals.
List available community services that support goal planning.	Providing cues	
Describe benefits of empowerment.	Discussion; Modeling	1. Group discussion about definition and benefits of empowerment.<br>2. Facilitators share their own empowerment stories.<br>3. Government information centers present information about relevant government services with Q&A session.
Describe process necessary to use government subsidies, schemes, and other resources.	Discussion	
Recognize positive versus negative interviewing methods.	Discussion	1. Facilitators use a mock “good” and “bad” interview to stimulate discussion contrasting effective and ineffective interviewing methods.
Practice developing a CV.	Feedback	1. Participants develop CVs with direct feedback from facilitators.

<table>
<tr><td colspan="3"><u>Module 5 PO</u>: Participants develop enhanced sexual communication and sexual and reproductive health knowledge.</td></tr>
<tr><td>Change Objectives</td><td>Methods</td><td>Applications</td></tr>
<tr><td>Name the parts and functions of the male and female sexual and reproductive health systems.</td><td rowspan="4">Active learning</td><td rowspan="4">1. Medical officer-led lecture on male and female reproductive anatomy and physiology (including sexual and reproductive misconceptions that have been linked to DV perpetration, menstruation, conception, pregnancy, and reproductive health services) with Q&A session.</td></tr>
<tr><td>Explain the process of menstruation and conception.</td></tr>
<tr><td>Identify misconceptions about reproductive health issues that often result in domestic violence.</td></tr>
<tr><td>List pregnancy support services.</td></tr>
<tr><td>Identify misconceptions about sex and sexuality.</td><td>Belief selection, Discussion</td><td>1. Individual quiz about common reproductive and sexual health issues used to facilitate discussion to clarify misconceptions regarding sex and sexuality.</td></tr>
<tr><td>List means of increasing romance.</td><td>Active learning</td><td>1. Participants play board game that introduces means of increasing romance in relationship.</td></tr>
<tr><td>Discuss the importance of understanding partner’s sexual expectations and concerns.</td><td rowspan="2">Framing; Consciousness-raising</td><td rowspan="2">1. Medical officer-led lecture about sexual communication (including discussing sexual expectations with partner) and respecting sexual partner (including consent).</td></tr>
<tr><td>Describe what constitutes a good sexual partner.</td></tr>
<tr><td>Practice effective sexual communication</td><td>Active learning</td><td>1. Practice sexual communication with partner through take-home exercise.</td></tr>
<tr><td colspan="3"><u>Module 6 PO:</u> Participants’ definitions of behaviors constituting IPV will expand and will be less accepting of IPV</td></tr>
<tr><td>Change Objectives</td><td>Methods</td><td>Applications</td></tr>
<tr><td>Define comprehensively behaviors constituting DV.</td><td>Discussion</td><td rowspan="3">1. Competitive listing of examples of DV in pairs
2. Participants individually distribute pebbles in glasses labeled with different examples of DV based on perceived severity of abuse. Facilitator uses group distributions of pebbles to lead discussion to facilitate expansion of definition of DV.
3. Facilitators lead discussion regarding effects of DV on victim, perpetrator, children and family
4. Discussion about environmental cues that perpetuate DV acceptance and the need to challenge them.</td></tr>
<tr><td>Describe the effects of DV on the survivor, perpetrator, family, and children.</td><td>Personalize risk</td></tr>
<tr><td>Critique the belief that violence is situationally acceptable and useful.</td><td>Resistance to social pressure</td></tr>
</table>

Table 2. Theory-informed Methods and Practical Applications
